# Amplified Fluorescent Aptasensor for Ochratoxin A Assay Based on Graphene Oxide and RecJ_f_ Exonuclease

**DOI:** 10.3390/toxins12110670

**Published:** 2020-10-23

**Authors:** Han Zhao, Dehui Xiong, Ying Yan, Changbei Ma

**Affiliations:** School of Life Sciences, Central South University, Changsha 410013, China; zhaohan202010@163.com (H.Z.); xiongdehui@csu.edu.cn (D.X.); Yany2018@csu.edu.cn (Y.Y.)

**Keywords:** fluorescence, ochratoxin A, RecJ_f_ exonuclease, graphene oxide, signal amplification

## Abstract

In this study, we developed an aptamer-based fluorescent sensing platform for the detection of ochratoxin A (OTA) based on RecJ_f_ exonuclease-assisted signal amplification and interaction between graphene oxide (GO) and the OTA aptamer (OTA-apt). After optimizing the experimental conditions, the present aptamer-based sensing system can exhibit excellent fluorescent response in the OTA assay, with a limit of detection of 0.07 ng/mL. In addition to signal amplification, this strategy is also highly specific for other interfering toxins. Furthermore, this aptasensor can be reliably used for assessing red wine samples spiked with different OTA concentrations (2.4, 6 and 20 ng/mL). The proposed assay plays an important role in the field of food safety and can be transformed for detecting other toxins by replacing the sequence that recognizes the aptamer.

## 1. Introduction

Ochratoxin A (OTA), a common mycotoxin, is a toxic secondary metabolite in fungi mainly produced by *Aspergillus ochraceus, Aspergillus carbonarius,* and *Penicillium verrucosum* [1]. Foodstuffs such as grains, coffee, cocoa, nuts, spices, wine, meat products, and dried fruits, are vulnerable to OTA contamination [2,3]. Toxicological studies have shown that OTA adversely affects humans as it may cause hepatotoxicity, nephrotoxicity, immunotoxicity, carcinogenicity, and teratogenicity [4]. In addition, excessive OTA in the human body can passively affect certain important biochemical processes; for example, it may increase lipid peroxidation, damage saccharide and calcium metabolism, and destroy mitochondrial function [5,6]. Removal of OTA at high temperature is challenging, and it has a half-life up to 35.5 days, which leads to its accumulation in the human body [7]. Owing to its potential threat to human health, OTA has been considered as a human carcinogen (Group 2B) by the International Agency for Research on Cancer (IARC), and the European Commission has stipulated that the maximum content of OTA in raw grains and soluble coffee is 5 µg/kg and 10 µg/kg, respectively [1,8]. To eliminate the possible risk of OTA accumulation, efficient and sensitive assays for OTA quantitation should be urgently developed.

Traditionally, several analytical methods, including mass spectrometry (MS) [9], gas chromatography (GC) [10], thin-layer chromatography (TLC) [11], and high-performance liquid chromatography (HPLC) [12] have been used for OTA determination. Although these conventional methods exhibit low detection limits and high selectivity, the disadvantages are obvious, such as the requirement of multiple sample pretreatment, personnel training, and time-consuming operation. Recently, immunoassays, such as the enzyme-linked immunosorbent assay (ELISA), were developed as an alternative method for OTA analysis, which possesses the advantages of simplicity and sensitivity [13,14]. Nevertheless, ELISA involves complicated washing steps and the preparation of expensive antibodies. In addition, antibodies are susceptible to environmental conditions such as pH, ionic strength, and temperature, which significantly restrict the range of application. Thus, high-efficiency and low-cost methods should be developed for detecting OTA. 

Aptamers are short oligonucleotides or peptide molecules, screened using an in vitro selection process called systematic evolution of ligands by exponential enrichment (SELEX) [15,16,17,18,19,20]. They can form unique secondary structures, which can specifically bind to their target molecules such as small molecules, proteins, amino acids, cells, and tissues with high affinity [21,22,23]. Compared to antibodies, aptamers exhibit the following advantages. First, they can be easily chemically modified because of their small molecular weight. Second, highly stable aptamers with prominent target diversity can be easily synthesized at low cost. Owing to these outstanding merits, several aptamer-based methods for OTA determination have been developed, such as electrochemistry- [24,25], fluorescence- [26,27], and colorimetry-based assays [28,29]. The first OTA aptamer synthesized by Cruz-Aguado for OTA detection was reported in 2008 and this aptamer has been widely used in the construction of many biosensors [30]. For example, Kuang et al. established an aptamer-based electrochemical strategy for detecting OTA [31]. Despite showing higher sensitivity, its performance was limited by long analysis time and the use of tedious electrode preparation steps. Lee et al. proposed a colorimetric aptamer sensor for OTA determination based on the formation of G-quadruplex DNAzymes [29]. However, this strategy has relatively low sensitivity. Analysis based on fluorescence has attracted widespread attention because of facile operation process, high sensitivity, and low production cost. Nonetheless, the aptamer and targeted molecule binding ratio is 1:1 in these methods, which limits signal amplification. Hence, fluorescence-based methods that can result in signal amplification are urgently required. 

As a derivative of graphene, graphene oxide (GO) has gained considerable attention due to its special optical, electronic, and thermal properties, and aqueous dispersibility [32]. GO can adsorb single-stranded DNA (ssDNA) via hydrophobic interactions, hydrogen bonding, and π-stacking interactions, whereas rigid double-stranded DNA (dsDNA) or well-folded DNA cannot be adsorbed on the surface of GO [33,34]. Based on these characteristics, GO coupled with DNA-based detection strategies has been successfully utilized for the detection of several targets [35,36]. 

In this study, we propose an aptamer-based fluorescent strategy that involves RecJ_f_ exonuclease (RecJ_f_ exo)-triggered signal amplification on a GO platform. The high affinity of OTA aptamer (OTA-apt) against OTA can hinder the adsorption on GO and subsequently initiate digestion with RecJ_f_ exo. The amplified fluorescent signal is obtained by coupling RecJ_f_ exo with the catalytic recycling of OTA. The proposed sensing platform is highly sensitive and selective for OTA and can be successfully used for OTA analysis in real samples.

## 2. Results and Discussion

### 2.1. Principle of the OTA Assay 

**Figure 1:**
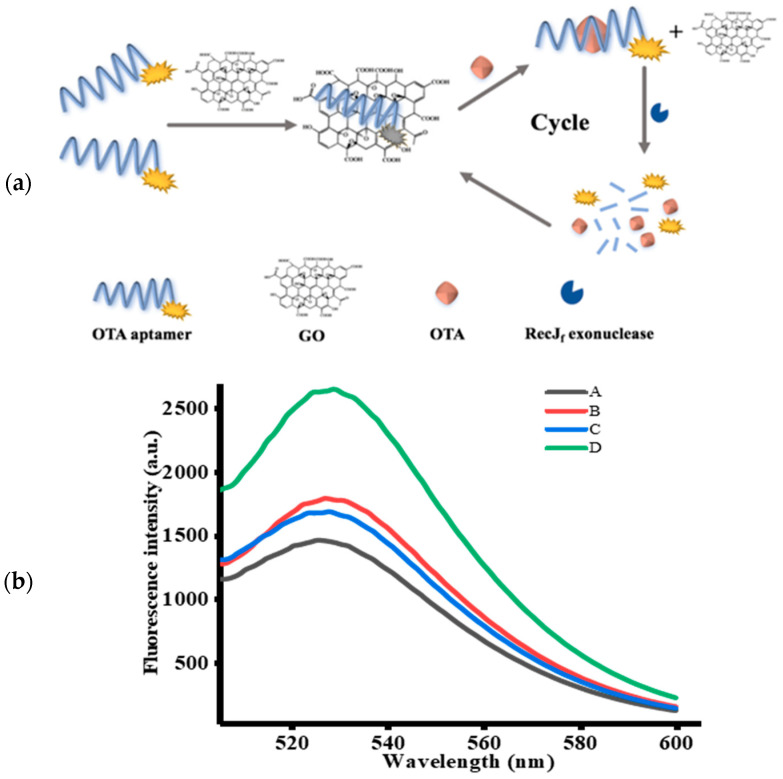
(**a**) Schematic illustration of RecJ_f_ exo-assisted signal amplification strategy for ochratoxin A (OTA) detection. (**b**) Fluorescence emission spectra of FAM-labeled OTA-apt under different cases (*n* = 3). (A) OTA-apt + graphene oxide (GO), (B) OTA + OTA-apt + GO, (C) OTA-apt + GO + RecJ_f_ exo, (D) OTA + OTA-apt + GO + RecJ_f_ exo. The concentrations of OTA, OTA-apt, GO, and RecJ_f_ exo were 400 ng/mL, 300 nM, 10 μg/mL, and 0.06 U/μL, respectively.

In this study, a sensitive and highly efficient aptamer-based fluorescent signal amplification strategy for OTA detection based on GO and RecJ_f_ exo was proposed, the mechanism of which is illustrated in Figure 1a. OTA-apt shows remarkable fluorescence owing to the presence of carboxyfluorescein (FAM) at its 5′ end. In the absence of OTA, FAM-labeled OTA-apt can be adsorbed on GO surface via π-stacking interactions between nucleobases and the GO surface, and the electrostatic driving force. The conjugation of OTA-apt to GO can effectively prevent the OTA-apt from undergoing RecJ_f_ exo cleavage. Consequently, a quenching fluorescence signal was observed. On the contrary, OTA can specifically bind to its target OTA-apt, inducing changes in the conformation of OTA-apt from ssDNA to antiparallel G-quadruplex, which cannot be adsorbed on the GO surface [37]. The free OTA-apt can be hydrolyzed by RecJ_f_ exo based on its ability to remove monodeoxynucleotides from ssDNA in the 5′–3′ direction, thereby releasing the FAM fluorophore and OTA [38]. As a result, a strong fluorescence signal was obtained. The freed OTA can bind with another OTA-apt to initiate a new cycle of cleavage reaction. Using this RecJ_f_ exo-assisted signal amplification strategy, an increasing amount of OTA-apt can be digested with a low amount of OTA, which significantly increases the detection sensitivity. Thus, on the basis of the changes in fluorescence signal, quantitative and sensitive analysis of OTA can be realized. 

### 2.2. Feasibility of the Designed Strategy

To confirm the feasibility of this aptasensor, the fluorescence emission spectrum of FAM-labeled OTA-apt under different circumstances was studied. Firstly, we investigated the variation in fluorescence intensity without RecJ_f_ exo. As can be seen from Figure 1b, a lower fluorescence signal was measured (curve A) with the introduction of OTA-apt and GO, indicating that the fluorescence of FAM was quenched by GO. Previous reports have shown that graphene can be used as a good quencher of organic dyes based on fluorescence resonance energy transfer effect (FRET) from dyes to graphene. Conversely, as depicted in curve B, a prominent fluorescence enhancement was observed upon addition of OTA. This phenomenon indicated that the high affinity of OTA-apt against OTA can induce the formation of OTA/OTA-apt complex, which is a steady antiparallel G-quadruplex structure that can be desorbed from the GO surface and can hinder the FRET from FAM to GO. We further investigated the signal amplification strategy using RecJ_f_ exo. In this condition, only a slight increase in background fluorescence (curve C) was monitored compared to curve A without OTA. Nevertheless, in the presence of OTA and RecJ_f_ exo, the fluorescence intensity obtained from curve D was stronger than that in the absence of RecJ_f_ exo (curve B), which was attributed to RecJ_f_ exo-catalyzed hydrolysis of OTA-apt and the release of OTA and the fluorophore. The obtained OTA can combine with another OTA-apt to initiate a new cycle, and, as this proceeds, the amount of fluorophores generated increases. Thus, a small amount of OTA can lead to the release of a considerable amount of fluorophores, dramatically amplifying the fluorescence signal. Above all, these intriguing results suggest that this signal-amplifying aptasensor can be successfully applied to OTA assay.

### 2.3. Optimization of Experimental Conditions

**Figure 2:**
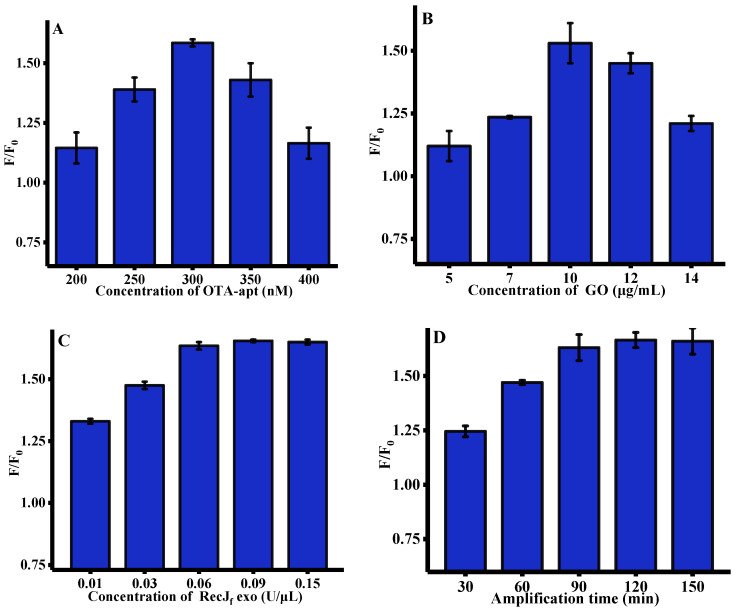
Optimization of experimental conditions for OTA assay (*n* = 3). (**A**) Concentration of OTA-apt, (GO) = 10 μg/mL, RecJ_f_ exo = 0.2 U/μL, (Amplification time) = 90 min, (OTA reaction time) = 15 min; (**B**) concentration of GO, (OTA-apt) = 300 nM, (RecJ_f_ exo) = 0.2 U/μL, (Amplification time) = 90 min, (OTA reaction time) = 15 min; (**C**) concentration of RecJ_f_ exo, (OTA-apt) = 300 nM, (GO) = 10 μg/mL, (Amplification time) = 90 min, (OTA reaction time) = 15 min; (**D**) amplification time, (OTA-apt) = 300 nM, (GO) = 10 μg/mL, (RecJ_f_ exo) = 0.06 U/ μL, (OTA reaction time) = 15 min. F_0_ and F were the fluorescence intensities in the absence and presence of OTA. (OTA) = 500 ng/mL, (excitation wavelength) = 490 nm, (emission wavelength) = 525 nm. Error bars represented the standard deviation of three experiments.

To acquire a desirable sensing performance, several pivotal parameters, such as the concentration of OTA-apt, GO, and RecJ_f_ exo, and amplification time, which may affect fluorescent response, were optimized. As depicted in Figure 2A, with the concentration of OTA-apt gradually increased from 200 to 300 nm, the enhanced fluorescence signal can be observed. However, the fluorescence intensity was gradually quenched after 300 nM. We speculated that excessive OTA-apt cannot be absorbed on the GO surface, leading to an increase in background fluorescence (F_0_). Therefore, 300 nM OTA-apt was selected for OTA analysis in subsequent work. The concentration of GO, which is closely related to its ability to adsorb OTA-apt, was further optimized and the results are shown in Figure 2B. The fluorescence response to OTA increased with the concentration of GO up to 10 μg/mL and then decreased drastically. This is mainly due the fact that excess GO may adsorb OTA/OTA-apt complex. Thus, 10 μg/mL of GO was used throughout the experiment. RecJ_f_ exo, which is a single-strand-specific exonuclease, plays a crucial role in this signal amplification strategy and its concentration was also optimized. Figure 2C shows that the ratio of fluorescence intensity increased significantly with the concentration of RecJ_f_ exo and approached a maximum at 0.06 U/μL, indicating that the digestion process had reached saturation. Finally, the effect of RecJ_f_ exo amplification time on fluorescence signal was researched. As shown in Figure 2D, the relative fluorescence intensity peaked after 90 min, indicating the completion of OTA-apt hydrolysis by RecJ_f_ exo.

### 2.4. Quantitative Determination of OTA

**Figure 3:**
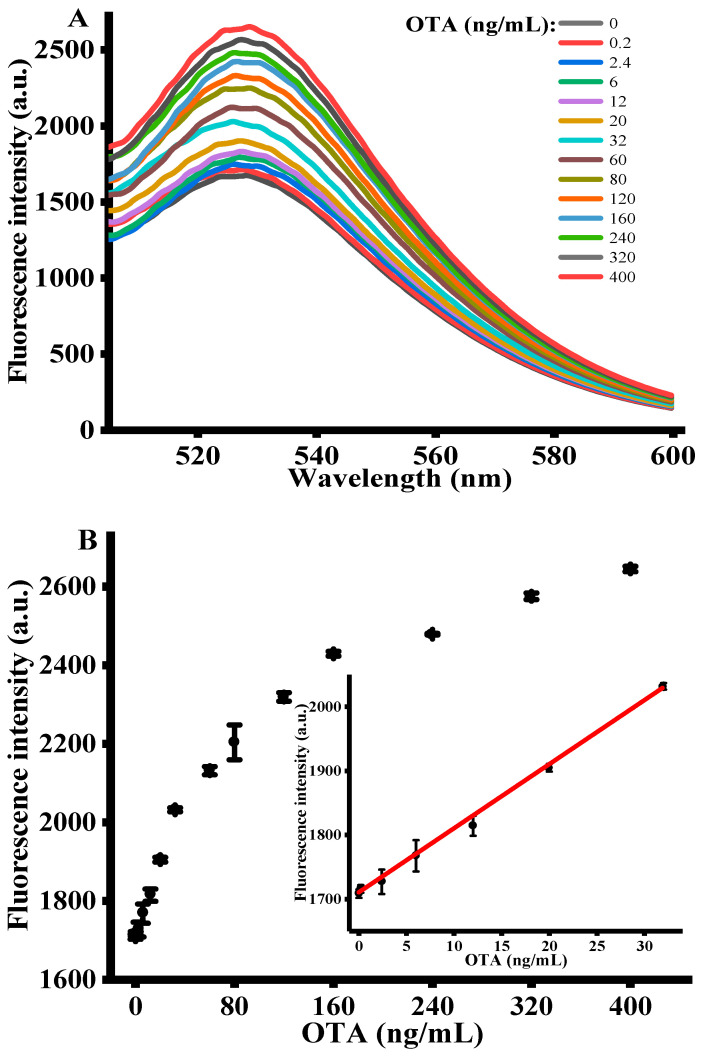
(**A**) Fluorescent emission spectra with different concentrations of OTA (*n* = 3). (**B**) The relationship between the fluorescence intensity and OTA concentration. (OTA-apt) = 300 nM, (GO) = 10 μg/mL, (OTA reaction time) = 15 min, (RecJ_f_ exo) = 0.06 U/μL, (Amplification time) = 90 min, (excitation wavelength) = 490 nm, (emission wavelength) = 525 nm. Error bars were obtained from three replicated measurements.

Under the optimized experimental conditions, the sensing performance of signal amplification strategy was assessed by adding various concentrations of OTA (i.e., 0–400 ng/mL) into the reaction system. Figure 3A shows the fluorescence emission spectra of FAM-labeled OTA-apt with different concentrations of OTA. It is apparent that the fluorescence emission intensity at 525 nm proportionally raised with OTA concentrations and plateaued at 400 ng/mL. This demonstrated that the antiparallel G-quadruplex structure formed by the specific binding of OTA with OTA-apt cannot be adsorbed on GO surface, resulting in a high fluorescence. The inset of Figure 3B shows the calibration curve for quantitative detection of OTA. A good linear relationship between fluorescence intensity and OTA concentration was observed with the dynamic range from 0.2 to 32 ng/mL. Using the origin software calculation, the linear regression equation obtained was F = 10.001 C + 1709.43 with a correlation coefficient of 0.999, where C was the concentration of OTA (ng/mL) and F represented peak intensity. The limit of detection (LOD) was accessed to be 0.07 ng/mL in accordance with the 3σ rule. In comparison with previous sensing platforms listed in Table 1, the detection limit of our strategy is superior than that of colorimetric method, carbon nanohorns-based fluorescence method, SYBR gold-based fluorescence method and copper nanoparticles-based fluorescence method. Despite many advantages in colorimetric assay, such as wider linear range, they are still affected by comparatively lower sensitivity and anti-interference ability. Electrochemical methods exhibit excellent detection performance, but the process of sensor fabrication is complicated, and the analysis process is time-consuming [39,40]. What is more, compared with the other fluorescent method shown in Table 1, the present work we proposed exhibits higher sensitivity, stability and reproducibility [3,41,42]. Hence, we concluded that this sensing strategy can be used for OTA determination with high sensitivity. At the same time, this fluorescence-based aptasensor can be further utilized for the other toxins analysis by replacing the specific sequence of the aptamer [43]. 

### 2.5. Evaluation of Specificity 

**Figure 4:**
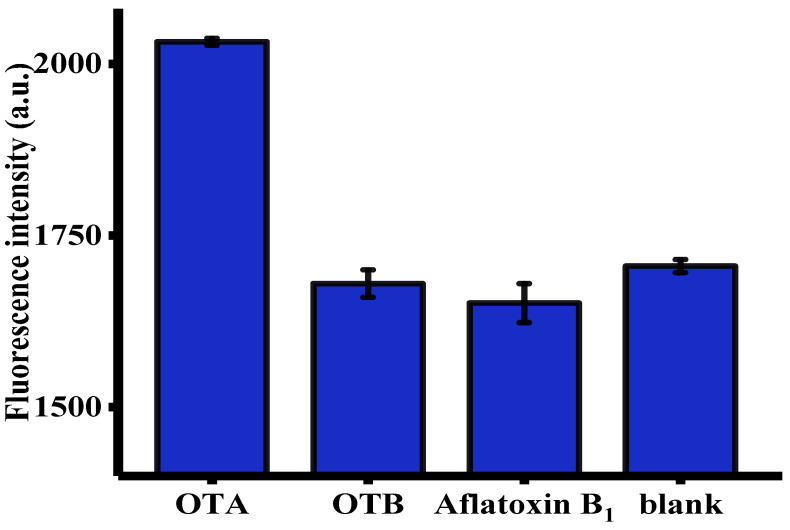
Selectivity of the signal amplification of the aptasensor toward OTA, OTB, and aflatoxin B_1_ (AFB_1_) (*n* = 3). The concentration of OTA was 32 ng/mL. The concentration of OTB and AFB_1_ were both of 100 ng/mL. Error bars were obtained from three replicated measurements.

The selectivity of this fluorescent aptasensor towards OTA was evaluated using OTB and aflatoxin B_1_ (AFB_1_) as controls [6]. As shown in Figure 4, only OTA, but not other toxins, such as OTB and AFB_1_, generated an intense fluorescence enhancement. This was primarily attributed to unique specificity of OTA-apt toward OTA [44]. These satisfactory results were indicative of the selectivity of this method and the broad prospects for its practical application in real samples.

### 2.6. OTA Assay in Red Wine Samples

In order to verify the applicability and accuracy of this fluorescent aptasensor for the analysis of red wine samples, recovery experiments for OTA determination were performed in 20-fold diluted red wine samples [38]. The corresponding results are displayed in Table 2. The fluorescence signal recovery was 93.8, 97.3, and 106.6% in the samples spiked with 2.4, 6, and 20 ng/mL of OTA, respectively, confirming the viability of this method for OTA assay in drink samples.

## 3. Conclusions

In general, a fluorescence-based aptasensor was designed for ultrasensitive determination of OTA, which relied on OTA-induced release of OTA-apt from the GO surface and RecJ_f_ exo-catalyzed cyclic signal amplification. We suggest that this signal amplification strategy exhibited several merits. First, this method showed high sensitivity, with an extremely low detection limit of 0.07 ng/mL, due to RecJ_f_ exo-assisted target recycling amplification. In addition, studies on the effect of certain interfering mycotoxins on the detection platform have confirmed its excellent selectivity. In addition, this sensing strategy has been successfully applied for OTA assay in real samples with encouraging results. This signal amplified aptasensor can be modified for the detection of a wide range of targets by using their corresponding aptamers. Thus, this method may find an extensive application prospect in the fields of food hygiene and environmental science.

## 4. Materials and Methods 

### 4.1. Materials and Reagents

Ochratoxin A (OTA) and ochratoxin B (OTB) were purchased from Pribolab Co., Ltd. (Qingdao, China). Aflatoxin B_1_ (AFB_1_) was obtained from Yuanye Co., Ltd. (Shanghai, China). Graphene oxide (GO) was bought from XFNANO Materials Tech Co., Ltd. (Nanjing, China). RecJ_f_ exonuclease (RecJ_f_ exo) and 10 × NEBuffer 2 (10 mM Tris-HCl, 50 mM NaCl, 10 mM MgCl_2_, 1 mM DTT, pH 7.9) were ordered from Takara Biotechnology Co., Ltd. (Dalian, China). The FAM-labeled DNA probe was synthesized by Sangon Biotechnology Co., Ltd. (Shanghai, China). The DNA sequence was dissolved in TE buffer and stored at −20 °C for further use. The sequence of DNA probe was listed as follows: 5′-FAM-GATCGGGTGTGGGTGG CGTAAAGGGAGCATCGGACA-3′. Ultrapure water (18.2 MΩ.cm) processed with a Milli-Q water purification system (Millipore Corp, Bedford, MA, USA) was used in the whole experiment.

### 4.2. Apparatus

Fluorescent emission spectra and fluorescence intensities were measured using an F-2700 fluorescence spectrophotometer (Hitachi, Japan). The excitation wavelength was set at 490 nm at room temperature and emission spectra were recorded in the wavelength of 505–600 nm with slit widths of emission and excitation both set at 5 nm.

### 4.3. Optimization of Reaction Conditions

To obtain the best experimental performance, several reaction parameters were first optimized, including the concentrations of OTA aptamer (OTA-apt), GO, and RecJ_f_ exo, and amplification time. The OTA-apt concentration ranged from 200 nM to 400 nM. The GO concentration used was 5–14 μg/mL. The concentration of RecJ_f_ exo used was 0.0–0.15 U/μL. The amplification time was 30–150 min. 

### 4.4. Fluorescent Detection of OTA

OTA was detected quantitatively as described below. First, a series of OTA concentrations (0–400 ng/mL) and 300 nM OTA-apt with the FAM fluorophore were added into the 1× NEBuffer 2 (10 mM Tris-HCl, 50 mM NaCl, 10 mM MgCl_2_, 1 mM DTT, pH 7.9). The mixture was incubated for 15 min at 25 °C. Subsequently, 10 μg/mL GO was added and allowed to incubate at 25 °C for 15 min. Afterwards, 0.06 U/μL RecJ_f_ exo was added for the digestion reaction in a final volume of 100 μL. After incubation for 90 min at 37 °C, the fluorescence intensity was recorded at room temperature using a F-2700 fluorescence spectrophotometer.

### 4.5. OTA Assay in Red Wine Samples

To verify the feasibility and reliability of the proposed protocol for OTA analysis in practical applications, red wine, which was purchased from a local supermarket, was utilized as the actual sample. Prior to the recovery experiment, the wine sample was filtered to remove the sediment and diluted with buffer solution. Then, 2.4, 6, and 12 ng/mL OTA were spiked into 20-fold diluted red wine samples. The samples of different OTA concentrations were measured three times, respectively. The subsequent reaction process and fluorescent measurements were the same as that described above.

## Figures and Tables

**Table 1:** Comparison with different reported methods for OTA detection.

Method	Material	LOD (ng/mL)	Dynamic Range (ng/mL)	Reference
Colorimetry	Gold nanorods	44	4–80	[28]
Colorimetry	G-quadruplex DNAzymes	0.4	--	[29]
Electrochemistry	Magnetic nanoparticles	0.01	0.01–5	[39]
Electrochemistry	Polythiophene-3-carboxylic acid	0.125	0.125–2.5	[40]
Fluorescence	Carbon nanohorns	1.6	4–400	[3]
Fluorescence	SYBR gold	4.7	8–1000	[41]
Fluorescence	Copper nanoparticles	5	0–100	[42]
Fluorescence	Graphene oxide	0.07	0.2–32	This work

**Table 2:** Application of aptasensor for OTA detection in red wine.

Sample	Added (ng/mL)	Found (ng/mL)	Recovery (%)
1	2.4	2.25 ± 0.28	93.8
2	6	5.84 ± 0.46	97.3
3	20	21.31 ± 0.32	106.6

## References

[1] Van der Merwe K.J., Steyn P.S., Fourie L., Scott D.B., Theron J.J. (1965). Ochratoxin A, a toxic metabolite produced by Aspergillus ochraceus Wilh. Nature.

[2] Covarelli L., Beccari G., Marini A., Tosi L. (2012). A review on the occurrence and control of ochratoxigenic fungal species and ochratoxin A in dehydrated grapes, non-fortified dessert wines and dried vine fruit in the Mediterranean area. Food Control..

[3] Wu H., Liu R.J., Kang X.J., Liang C.Y., Lv L., Guo Z.G. (2017). Fluorometric aptamer assay for ochratoxin A based on the use of single walled carbon nanohorns and exonuclease III-aided amplification. Mirochim. Acta.

[4] Pfohl-Leszkowicz A., Manderville R.A. (2007). Ochratoxin A: An overview on toxicity and carcinogenicity in animals and humans. Mol. Nutr. Food Res..

[5] Costa J.G., Saraiva N., Guerreiro P.S., Louro H., Silva M.J., Miranda J.P., Castro M., Batinic-Haberle I., Fernandes A.S., Oliveira N.G. (2016). Ochratoxin A-induced cytotoxicity, genotoxicity and reactive oxygen species in kidney cells: An integrative approach of complementary endpoints. Food Chem. Toxicol..

[6] Wu K.F., Ma C.B., Zhao H., Chen M.J., Deng Z.Y. (2019). Sensitive aptamer-based fluorescene assay for ochratoxin A based on RNase H signal amplification. Food Chem..

[7] Studer-Rohr I., Schlatter J., Dietrich D.R. (2000). Kinetic parameters and intraindividual fluctuations of ochratoxin A plasma levels in humans. Arch. Toxicol..

[8] Wei Y., Zhang J., Wang X., Duan Y.X. (2015). Amplified fluorescent aptasensor through catalytic recycling for highly sensitive detection of ochratoxin A. Biosens. Bioelectron..

[9] Reinsch M., Töpfer A., Lehmann A., Nehls I., Panne U. (2007). Determination of ochratoxin A in beer by LC–MS/MS ion trap detection. Food Chem..

[10] Olsson J., Börjesson T., Lundstedt T., Schnürer J. (2002). Detection and quantification of ochratoxin A and deoxynivalenol in barley grains by GC-MS and electronic nose. Int. J. Food Microbiol..

[11] Pittet A., Royer D. (2002). Rapid, low cost thin-layer chromatographic screening method for the detection of ochratoxin A in green coffee at a control level of 10 microg/kg. J. Agric. Food Chem..

[12] Tessini C., Mardones C., von Baer D., Vega M., Herlitz E., Saelzer R., Silva J., Torres O. (2010). Alternatives for sample pre-treatment and HPLC determination of Ochratoxin A in red wine using fluorescence detection. Anal. Chim. Acta.

[13] Yu F.Y., Chi T.F., Liu B.H., Su C.C. (2005). Development of a sensitive enzyme-linked immunosorbent assay for the determination of ochratoxin A. J. Agric. Food Chem..

[14] Liu B.H., Tsao Z.J., Wang J.J., Yu F.Y. (2008). Development of a monoclonal antibody against ochratoxin A and its application in enzyme-linked immunosorbent assay and gold nanoparticle immunochromatographic strip. Anal. Chem..

[15] Tuerk C., Gold L. (1990). Systematic evolution of ligands by exponential enrichment: RNA ligands to bacteriophage T4 DNA polymerase. Science.

[16] Ellington A.D., Szostak J.W. (1990). In vitro selection of RNA molecules that bind specific ligands. Nature.

[17] Zhou J., Battig M.R., Wang Y. (2010). Aptamer-based molecular recognition for biosensor development. Anal. Bioanal. Chem..

[18] Dai S., Wu S., Duan N., Wang Z. (2016). A luminescence resonance energy transfer based aptasensor for the mycotoxin Ochratoxin A using upconversion nanoparticles and gold nanorods. Microchim. Acta.

[19] Huo Y., Qi L., Lv X.J., Lai T., Zhang J., Zhang Z.Q. (2016). A sensitive aptasensor for colorimetric detection of adenosine triphosphate based on the protective effect of ATP-aptamer complexes on un- modified gold nanoparticles. Biosens. Bioelectron..

[20] Lv L., Li D.H., Cui C.B., Zhao Y.Y., Guo Z.J. (2017). Nuclease-aided target recycling signal amplification strategy for ochratoxin A monitoring. Biosens. Bioelectron..

[21] Liu H.S., Ma C.B., Ning F., Chen H.C., He H.L., Wang K.M., Wang J. (2017). A facile label-free G-quadruplex based fluorescent aptasensor method for rapid detection of ATP. Spectrochim. Acta A Mol. Biomol. Spectrosc..

[22] Chen M.J., Tang Z.W., Ma C.B., Yan Y. (2020). A fluorometric aptamer based assay for prostate specific antigen based on enzyme-assisted target recycling. Sens. Actuators B Chem..

[23] Xia X., Wang Y., Yang H., Dong Y., Zhang K., Lu Y., Deng R., He Q. (2019). Enzyme-free amplified and ultrafast detection of aflatoxin B-1 using dual-terminal proximity aptamer probes. Food Chem..

[24] Wu J.J., Chu H.Q., Mei Z.L., Deng Y., Xue F., Zheng L., Chen W. (2012). Ultrasensitive one-step rapid detection of ochratoxin A by the folding-based electrochemical aptasensor. Anal. Chim. Acta.

[25] Sun A.L., Zhang Y.F., Sun G.P., Wang X.N., Tang D. (2017). Homogeneous electrochemical detection of ochratoxin A in foodstuff using aptamer-graphene oxide nanosheets and DNase I-based target recycling reaction. Biosens. Bioelectron..

[26] Zhao H., Xiang X.Y., Chen M.J., Ma C.B. (2019). Aptamer-Based Fluorometric Ochratoxin A Assay Based on Photoinduced Electron Transfer. Toxins.

[27] Wu K.F., Ma C.B., Zhao H., He H.L., Chen H.C. (2018). Label-Free G-Quadruplex Aptamer Fluorescence Assay for Ochratoxin A Using a Thioflavin T Probe. Toxins.

[28] Yu X.H., Lin Y.H., Wang X.S., Xu L.G., Wang Z.W., Fu F.F. (2018). Exonuclease-assisted multicolor aptasensor for visual detection of ochratoxin A based on G-quadruplex-hemin DNAzyme-mediated etching of gold nanorod. Microchim. Acta.

[29] Lee J., Jeon C.H., Ahn S.J., Ha T.H. (2014). Highly stable colorimetric aptamer sensors for detection of ochratoxin A through optimizing the sequence with the covalent conjugation of hemin. Analyst.

[30] Cruz-Aguado J.A., Penner G. (2008). Determination of ochratoxin a with a DNA aptamer. J. Agric. Food Chem..

[31] Kuang H., Chen W., Xu D.H., Xu L.G., Zhu Y.Y., Liu L.Q., Chu H.Q., Peng C.F., Xu C.L., Zhu S.F. (2010). Fabricated aptamer-based electrochemical “signal-off” sensor of ochratoxin A. Biosens. Bioelectron..

[32] Swathi R.S., Sebastian K.L. (2009). Long range resonance energy transfer from a dye molecule to graphene has (distance)(-4) dependence. J. Chem. Phys..

[33] Toda K., Furue R., Hayami S. (2015). Recent progress in applications of graphene oxide for gas sensing: A review. Anal. Chim. Acta.

[34] Lee J., Kim Y.K., Min D.H. (2011). A new assay for endonuclease/methyltransferase activities based on graphene oxide. Anal. Chem..

[35] Chen J., Ge J., Zhang L., Li Z.H., Li J.J., Sun Y.J., Qu L.B. (2016). Reduced graphene oxide nanosheets functionalized with poly (styrene sulfonate) as a peroxidase mimetic in a colorimetric assay for ascorbic acid. Microchim. Acta.

[36] Chen M.J., Li W.K., Ma C.B., Wu K.F., He H.L., Wang K.M. (2019). Fluorometric determination of the activity of uracil-DNA glycosylase by using graphene oxide and exonuclease I assisted signal amplification. Microchim. Acta.

[37] Sheng L.F., Ren J.T., Miao Y.Q., Wang J.H., Wang E.K. (2011). PVP-coated graphene oxide for selective determination of ochratoxin A via quenching fluorescence of free aptamer. Biosens. Bioelectron..

[38] Yi H.Y., Xu W.J., Yuan Y.L., Wu Y.M., Chai Y.Q., Yuan R. (2013). A sensitive electrochemical aptasensor for thrombin detection based on exonuclease-catalyzed target recycling and enzyme-catalysis. Biosens. Bioelectron..

[39] Zamfir L.G., Geana I., Bourigua S., Rotariu L., Bala C., Errachid A., Jaffrezic-Renault N. (2011). Highly sensitive label-free immunosensor for ochratoxin A based on functionalized magnetic nanoparticles and EIS/SPR detection. Sens. Actuators B Chem..

[40] Zejli H., Goud K.Y., Marty J.L. (2018). Label free aptasensor for ochratoxin A detection using polythiophene-3-carboxylic acid. Talanta.

[41] Liu R.J., Wu H., Lv L., Kang X.J., Cui C.B., Feng J., Guo Z.J. (2018). Fluorometric aptamer based assay for ochratoxin A based on the use of exonuclease III. Microchim. Acta.

[42] Song C.X., Hong W.W., Zhang X.Y., Lu Y. (2018). Label-free and sensitive detection of Ochratoxin A based on dsDNA-templated copper nanoparticles and exonuclease-catalyzed target recycling amplification. Analyst.

[43] Xia X., Wang H., Yang H., Deng S., Deng R., Dong Y., He Q. (2018). Dual-Terminal Stemmed Aptamer Beacon for Label-Free Detection of Aflatoxin B-1 in Broad Bean Paste and Peanut Oil via Aggregation-Induced Emission. J. Agric. Food Chem..

[44] Wang S., Zhang Y.J., Pang G.S., Zhang Y.W., Guo S.J. (2017). Tuning the Aggregation/Disaggregation Behavior of Graphene Quantum Dots by Structure-Switching Aptamer for High-Sensitivity Fluorescent Ochratoxin A Sensor. Anal. Chem..

